# Engineering *Collariella virescens* Peroxygenase for Epoxides Production from Vegetable Oil

**DOI:** 10.3390/antiox11050915

**Published:** 2022-05-06

**Authors:** Dolores Linde, Alejandro González-Benjumea, Carmen Aranda, Juan Carro, Ana Gutiérrez, Angel T. Martínez

**Affiliations:** 1 Centro de Investigaciones Biológicas “Margarita Salas” (CIB), Consejo Superior de Investigaciones Científicas (CSIC), E-28040 Madrid, Spain; lolalinde@cib.csic.es (D.L.); jcarro@cib.csic.es (J.C.); 2 Instituto de Recursos Naturales y Agrobiología de Sevilla (IRNAS), Consejo Superior de Investigaciones Científicas (CSIC), E-41012 Seville, Spain; a.g.benjumea@irnas.csic.es (A.G.-B.); anagu@irnase.csic.es (A.G.); 3 Johnson Matthey, Cambridge Science Park U260, Cambridge CB4 0FP, UK; carmen.aranda@matthey.com

**Keywords:** unspecific peroxygenase (UPO), *Collariella virescens*, heme access channel, protein engineering, epoxidation, unsaturated fatty acids, sunflower oil, process optimization

## Abstract

Vegetable oils are valuable renewable resources for the production of bio-based chemicals and intermediates, including reactive epoxides of industrial interest. Enzymes are an environmentally friendly alternative to chemical catalysis in oxygenation reactions, epoxidation included, with the added advantage of their potential selectivity. The unspecific peroxygenase of *Collariella virescens* is only available as a recombinant enzyme (r*Cvi*UPO), which is produced in *Escherichia coli* for protein engineering and analytical-scale optimization of plant lipid oxygenation. Engineering the active site of r*Cvi*UPO (by substituting one, two, or up to six residues of its access channel by alanines) improved the epoxidation of individual 18-C unsaturated fatty acids and hydrolyzed sunflower oil. The double mutation at the heme channel (F88A/T158A) enhanced epoxidation of polyunsaturated linoleic and α–linolenic acids, with the desired diepoxides representing > 80% of the products (after 99% substrate conversion). More interestingly, process optimization increased (by 100-fold) the hydrolyzate concentration, with up to 85% epoxidation yield, after 1 h of reaction time with the above double variant. Under these conditions, oleic acid monoepoxide and linoleic acid diepoxide are the main products from the sunflower oil hydrolyzate.

## 1. Introduction

The oxirane ring of epoxides has been termed the “Lord of the chemical rings” [1] because of its high reactivity in the industrial production of bio-based chemicals and intermediates, including binder ingredients and resins. Epoxy resins comprise a group of cross-linkable materials, which polymerize with co-reactants (curing agents) into a matrix that can be used in a wide range of applications. Epoxy resins and curing agents usually contain more than one reaction site per molecule, to allow multiple crosslink reactions between them. Vegetable oils are one of the most important renewable feedstocks for a bio-based chemical industry [2,3,4]. The epoxides produced from oil fatty acids are possible ingredients for industrial resins (e.g., for board production), as long as they meet the required reaction selectivity and crosslinking properties.

**Figure 1:**
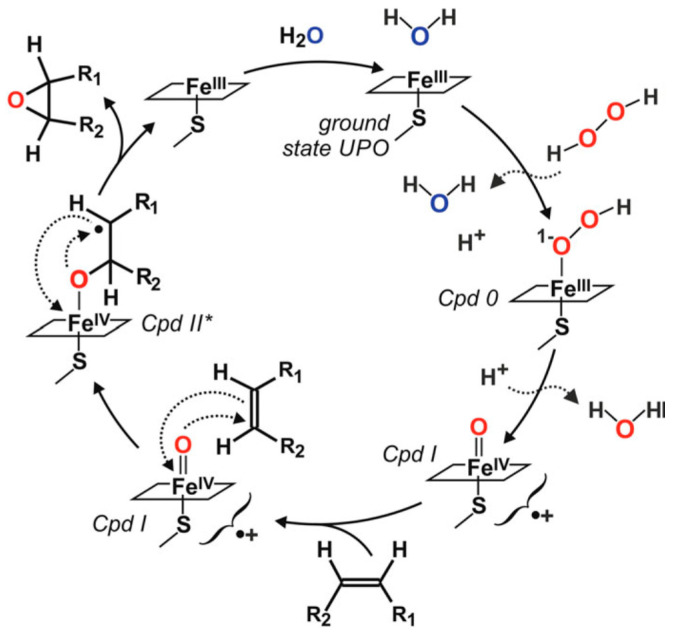
Proposed epoxidation cycle of UPO, showing activation of the ground state enzyme by H_2_O_2_, through hydroperoxo compound-0 (Cpd 0), to form reactive Cpd I able to epoxidize double bonds via a transient oxoferryl–substrate radical complex (Cpd II*). Reprinted from Ref. [19], 2020, Academic Press.

Fatty acid epoxidation is industrially performed by the Prileschajew reaction [5] via percarboxylic acids, traditionally generated by strong acids [6], but also using formic acid [7] or ion-exchange resin [8]. Attempts to use milder conditions include chemoenzymatic lipase–H_2_O_2_ reactions on oils, free fatty acids, and their methyl esters [9,10,11,12,13,14], which maintain the drawbacks due to the use of peracids and direct enzymatic epoxidation. Some plant peroxygenases [15], cytochrome P450 monooxygenases [16], and fungal unspecific peroxygenases (UPOs) [17] catalyze the direct epoxidation of (poly)unsaturated fatty acids. The latter enzymes present advantages related to their self-sufficiency (being independent of auxiliary proteins/modules and sources of reducing power) and secreted nature (being more stable than intracellular enzymes with monooxygenase activity) [18,19]. UPOs were known as aromatic peroxygenases [20], but, after first reports on their action on alkanes, fatty acids, and alcohols [21,22], numerous examples have shown their wide versatility on aliphatic compounds, including epoxidation reactions [23] and the name changed to unspecific peroxygenases (EC 1.11.2.1). The mechanism of this epoxidation reaction is illustrated in the catalytic cycle shown in Figure 1. This reaction is characterized by the presence of a modified compound II–substrate complex (Cpd II*), absent from the general peroxidase/peroxygenase catalytic cycle, that facilitates epoxide cyclization [19].

Recent studies on structure–function relationships in fatty-acid oxygenation by UPOs have revealed that the different peroxygenation patterns (enzyme regioselectivity) are ruled by the structure of the heme channel [24,25,26]. A good accessibility of the double bond of unsaturated fatty acids to the oxo group of the heme compound I (Cpd I formed after UPO activation by peroxide) is the key of the oxygenation reactions. In this way, different UPOs are able to produce different sets of oxygenated products (epoxy, hydroxy, and hydroxy–epoxy derivatives included) [17,21,27]. 

The UPO from the ascomycete *Collariella virescens* (syn. *Chaetomium virescens*; *Cvi*UPO) has been heterologously expressed in *Escherichia coli* and the recombinant enzyme (r*Cvi*UPO), isolated without any purification tag [28]. The obtained amounts are suitable for structure–function and (analytical scale) reaction optimization, making it a good starting point for future UPO studies. Moreover, r*Cvi*UPO shows good conversion of unsaturated fatty acids and, in contrast to other UPOs, generates epoxides as main products [25]. 

r*Cvi*UPO was already engineered by site-directed mutagenesis [25] in an initial attempt to mimic the heme environment of the UPO of related *Chaetomium globosum*, which efficiently epoxidizes unsaturated fatty acids [17]. Then, it was found that conversion of polyunsaturated omega 6 fatty acids (i.e., those with a double bond at the sixth position from the omega end) by the F88L variant of r*Cvi*UPO promoted diepoxidation [29]. This was explained by a wider heme access channel, facilitating epoxidation of two double bonds. Increased epoxidation selectivity was also reported for the I153T variant of the *Marasmius rotula* enzyme [26], the first UPO heterologously expressed in *E. coli* (r*Mro*UPO) [30]. However, r*Mro*UPO has the disadvantage, compared to r*Cvi*UPO, of its much lower expression in *E. coli* as a soluble enzyme. 

In the present work, we aimed to improve the conversion yield and selectivity of plant lipid epoxidation by r*Cvi*UPO via two different strategies: (i) enzyme engineering to achieve two epoxy groups per molecule, and (ii) analytical scale optimization of the reaction parameters to epoxidize hydrolyzed vegetable oil.

## 2. Materials and Methods

### 2.1. Production of Native Enzyme and Site-Directed Variants 

The *Cvi*UPO sequence [31] was optimized for *E. coli* expression (using the software Optimizer) [32], synthesized, cloned, and produced as previously described [28]. The non-mutated recombinant (hereinafter native) enzyme was purified by two chromatographic steps in an Äkta (GE Healthcare, Chicago, IL, USA) fast liquid chromatography system. The first step was cation exchange chromatography with a HiTrap SPFF column (GE, Healthcare, Chicago, IL, USA) in 10 mM Tris (pH 7.4). The proteins, eluted as a single peak (recorded at 420 nm) with a gradient of the same buffer supplemented with 1 M NaCl, were concentrated in an Amicon 3K device (Sigma-Aldrich, Saint Louis, MO, USA). The second step (to ensure protein purity) was size-exclusion chromatography with a Superdex 75 column (10/300 GL; GE Healthcare, Chicago, IL, USA) in 10 mM Tris (pH 7.4) with 0.15 M NaCl.

A molecular model of the *Cvi*UPO structure was obtained at the Swiss-Model server (http://swissmodel.expasy.org, accessed on 1 March 2022) [33] with the structure of the first-reported [34] and crystallized [35] UPO of *Agrocybe aegerita* (*Aae*UPO) as a template (PDB entry 2YP1). This model was used for the design of four mutated variants with progressively enlarged heme access channels. The F88A and T158A single mutations were introduced in the *Cvi*UPO gene cloned in pET23a using the Expand Long Template PCR kit from Roche (Basel, Switzerland) and the following oligonucleotides as primers (direct sequences with the mutated codons underlined): F88A: 5′-ACT TAC ACC GTT CAG CAG CGT ATC GCG AGT TAC GGT GAA ACG-3′, T158A: 5′-AAC CGC CAT AAC CTG GCG GAA CAT GAT GCA TCT C-3′. For double mutation, the vector containing the *Cvi*UPO gene with the first mutation was used as a template. The PCR products were digested with *Dpn*I and transformed into *E. coli* DH5α for propagation. The gene of the 6Ala sextuple variant—including the L64A, I61A, F88A, T157A, T158A, and T165A mutations—was synthesized by ATG-biosynthetics GmbH (Merzhausen, Germany). All the variants were produced in *E. coli* as active cytosolic enzymes, and purified as described above for the native r*Cvi*UPO.

Enzyme purity was confirmed under denaturing conditions by 12% polyacrylamide gel electrophoresis (PAGE) in the presence of 0.1% sodium dodecyl sulfate (SDS) and 1% mercaptoethanol [36]. Proper folding and binding of the cofactor were verified by analyzing the UV–visible spectrum of the resting state of the enzymes in 10 mM Tris (pH 7.4) using a Cary 60 spectrophotometer (Agilent, Santa Clara, CA, USA). Additionally, formation of the characteristic complex between reduced heme-thiolate enzymes (ferrous form) and carbon monoxide (CO) was assessed in 0.2 M phosphate (pH 8) after addition of Na_2_S_2_O_4_ and CO flushing. UPO concentrations were calculated using the r*Cvi*UPO molar extinction coefficient of ℇ_420_ = 114.2 mM^−1^·cm^−1^ [28].

### 2.2. Enzyme Kinetics

Optimal pH values for oxidation of several UPO substrates—namely veratryl and benzyl alcohols (10 mM) and naphthalene (1 mM) from Merck (Darmstadt, Germany) and 2,2′-azino-bis(3-ethylbenzothiazoline-6-sulfonic acid) (ABTS; 2 mM) from Boehringer Mannheim (Mannheim, Germany)—by the native r*Cvi*UPO and its variants were analyzed in the range of pH 2–10 using 0.2 M Britton–Robinson buffer, at 24 °C, in the presence of 1 mM H_2_O_2_. 

Kinetic curves for the above enzyme-reducing substrates were obtained (with spectrophotometric assays) from the initial (10–30 s) increase of product absorbance, using a Thermo Spectronic UV–visible spectrophotometer (Waltham, MA, USA) at the optimal pH for each enzyme and substrate. Oxidation of veratryl alcohol (0.09–50 mM, in 0.1 M acetate, pH 3 or 5) was followed at 310 nm (ℇ_310_ = 9300 M^−1^·cm^−1^), benzyl alcohol (0.3–50 mM, in 0.1 M Tris, pH 6) at 280 nm (ℇ_280_ = 1400 M^−1^·cm^−1^), naphthalene (0.03–2 mM, in 0.1 M tartrate, pH 6, with 5% ethanol) at 303 nm (ℇ_303_ = 2030 M^−1^·cm^−1^), and ABTS (0.04–5 mM in 0.1 M acetate, pH 4) at 436 nm (ℇ_436_ = 29,300 M^−1^·cm^−1^). Reactions (1 mL) were triggered by the addition of 1 mM or 24 mM H_2_O_2._ Kinetic curves for H_2_O_2_ were obtained with different peroxide concentrations (0.5–30 mM, in 0.1 M acetate, pH 4) in the presence of 2.5 mM ABTS whose (one-electron) oxidation was monitored at 436 nm (as explained above) for activity estimation (note that two moles of ABTS are oxidized by each mole of peroxide), and the H_2_O_2_ *K*_m_ values were obtained.

Kinetic curves for oleic acid oxidation (estimated chromatographically) were obtained by varying the concentration from 12.5 μM to 1.6 mM substrate, in 50 mM phosphate (pH 7) containing 20% acetone (*v*/*v*). Reactions (1 mL) were triggered by the addition of 24 mM H_2_O_2_ and stopped after 1 min by vigorous shaking with 100 μL of 0.1 M sodium azide. The oxygenated products were extracted, analyzed by gas chromatography–mass spectrometry (GC-MS) as described below, and their total abundance was used for the calculation of kinetic constants. All reactions were carried out in triplicate.

Curve-fit and data analysis for kinetic constant estimation were carried out using Sigma Plot 11.0. Michaelis–Menten constant (*K*_m_) and turnover number (catalytic constant, *k*_cat_), and their standard errors were obtained by non-linear fitting the *k*_obs_ values to Equation (1) (Michaelis–Menten model) except for (i) ABTS oxidation by rC*vi*UPO and T158A using 1 mM H_2_O_2_; (ii) ABTS oxidation by F88A (using either 1 mM or 24 mM H_2_O_2_); (iii) benzyl alcohol oxidation by F88A and F88A/T158A using 1 mM H_2_O_2_; (iv) oleic acid oxidation by the F88A, F88A/T158A, and 6Ala variants; (v) H_2_O_2_ reduction (in presence of 2.5 mM ABTS) by r*Cvi*UPO and T158A variant, where enzyme inhibition was observed, being therefore adjusted to Equation (2) (with the *k*_i_ inhibition constant being the concentration producing half maximum inhibition); and (vi) benzyl alcohol oxidation by r*Cvi*UPO and T158A using 1 mM H_2_O_2_ that was adjusted to Equation (3) (Hill model with *n*_H_ providing a measurement of the cooperativity of the substrate binding to the enzyme, and *K*_0.5_ being the substrate concentration for half saturation).

(1) $$f=\frac{k_{\mathrm{cat}\;}S}{K_{m}+S}$$

(2) $$f=\frac{k_{\mathrm{cat}}}{1+\frac{K_{m}}{S}+\frac{S}{k_{i}}}$$

(3) $$f=\frac{y_{0}+k_{\mathrm{cat}}S^{n_{H}}}{K_{0.5}{}^{n_{H}}+S^{n_{H}}}$$

### 2.3. Epoxidation of Individual Fatty Acids and Oil Hydrolyzate 

For evaluating the UPO epoxidation ability, the following 18-C unsaturated fatty acids (from Sigma-Aldrich, Saint Louis, MO, USA) were used as substrates: oleic (*cis*-9-octadecenoic, C18:1), linoleic (*cis*,*cis*-9,12-octadecadienoic, C18:2), and α-linolenic (*cis*,*cis*,*cis*-9,12,15-octadecatrienoic, C18:3) acids. Reactions running for 30 min were performed using 0.1 mM substrate, 0.25 or 0.4 µM enzyme (C18:1/C18:3 or C18:2 reactions, respectively), and 1.25 mM H_2_O_2_ in 50 mM phosphate (pH 7), at 30 °C in the presence of 20% acetone (for better substrate solubilization). For more complete characterization, epoxyoleic acid was prepared in larger scale (50 mL) reactions and purified by silica gel (60–200 µm) column chromatography using hexane–EtOAc (10:1 → 5:1) as the mobile phase.

Oil hydrolyzate was assayed for epoxide production as a more economical and sustainable substrate than pure fatty acids. Sunflower oil (supplied by Cargill in the frame of the project SusBind, https://susbind.eu, accessed on 1 March 2022) was saponified and the hydrolyzed fatty acids extracted at acidic pH as previously described [37]. Initial reactions were performed for 30 min using 0.1 mM hydrolyzate, 0.25 µM enzyme, and 1 mM H_2_O_2_ in 50 mM phosphate buffer (pH 7) containing 20% acetone (conditions similar to those used with individual fatty acids). In further reactions, up to 10 mM oil hydrolyzate was used, and the effect of several variables was studied including: pH 5.5/7.0, 20/30% acetone cosolvent, 0.25/0.50 µM enzyme (resulting in 100–400 substrate/enzyme (S/E) molar ratio), 1–100 mM H_2_O_2_ (resulting in 1.0–6.8 equivalents per fatty-acid double bond) added with a syringe pump, and 30/60 min reaction time. Given the results obtained with individual fatty acids, the F88A/T158A variant was selected for hydrolyzate reactions, compared with native r*Cvi*UPO. Epoxidation yields were calculated taking into account the epoxidation degree, the number of unsaturations, and the reaction conversion for each substrate. In all cases, control experiments were carried out under the same conditions (H_2_O_2_ included), but in the absence of enzyme. 

### 2.4. GC-MS Analyses

Products (and unreacted substrates) from the above reactions were liquid–liquid extracted with methyl *tert*-butyl ether (Sigma-Aldrich, Saint Louis, MO, USA), which was evaporated under a N_2_ stream. *N*,*O*-Bis(trimethylsilyl)trifluoroacetamide (Supelco, Bellefonte, PA, USA) was used to prepare trimethylsilyl derivatives. The GC-MS analyses were performed with an Agilent (Santa Clara, CA, USA) GC-MS QP2020 Ultra equipment using a fused-silica DB-5HT 30 m capillary column from J&W Scientific (Folsom, CA, USA). The oven was heated from 120 °C (1 min) to 300 °C (15 min) at 5 °C min^−1^. The injector and transfer line were kept at 300 °C. Compounds were identified by mass fragmentography and comparison of their mass spectra with those of authentic standards. Quantifications were obtained from total-ion peak areas (after deconvolution when partial overlapping was observed) and molar response factors of the same or similar compounds.

### 2.5. Chiral Analyses

Chiral analyses of epoxides of oleic acid were performed after derivatization into methyl esters (using trimethylsylyldiazomethane from Sigma-Aldrich, Saint Louis, MO, USA) with a Shimadzu (Kyoto, Japan) i-Prominence 2030C high-performance liquid chromatography (HPLC) equipment using an OB-H (250 × 4.6 cm, 5 μm particle) column from Daicel (Osaka, Japan). Compounds were eluted with hexane–^i^PrOH-AcOH (99.65:0.3:0.05), at a flow rate of 0.8 mL·min^−1^ and monitored at 202 nm. Enantiomer assignment was done following the reported literature [38].

## 3. Results and Discussion

### 3.1. Design and Catalytic Characterization of rCviUPO Variants

**Figure 2:**
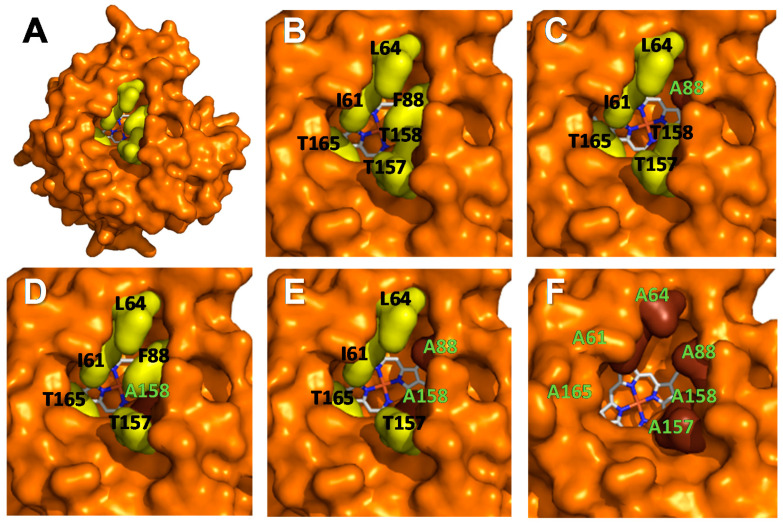
Solvent-access surface in *Cvi*UPO molecular model (**A**), and detail of the access channel to the heme cofactor (as CPK-colored sticks) in the native enzyme (**B**) and its F88A (**C**), T158A (**D**), F88A/T158A (**E**), and 6Ala (**F**) variants. Those residues to be mutated (black labels) have yellow surfaces; the already-introduced alanines (yellow labels in **C**–**F**) show brown surfaces.

Previous results with r*Cvi*UPO variants [25] led us to study the further broadening of its heme channel. For simplicity, the residues surrounding heme access were mutated into alanines; two simple (F88A and T158A), one double (F88A/T158A), and one sextuple (I61A/L64A/F88A/T157A/T158A/T165A) variants were designed (Figure 2). The variants were expressed as soluble active enzymes, and purified by a combination of cation exchange and size-exclusion chromatography [28]. In all cases, electrophoretic homogeneity was attained, as revealed by SDS-PAGE (Figure S1). Moreover, correct incorporation of the heme-thiolate group was confirmed by spectrophotometric analysis of the enzyme resting state, with the reduced enzyme complex with CO (Figure S2 main panels and insets, respectively) exhibiting characteristic maxima around 420 and 440 nm, respectively.

First, the optimal pH for oxidation of four peroxidase/peroxygenase substrates was determined for the native r*Cvi*UPO and variants (Figure S3). Kinetic curves and constants were first obtained with 1 mM H_2_O_2_ as the enzyme-oxidizing substrate (Figure S4A–D and Table S1, respectively). Catalytic constants for H_2_O_2_ were determined using 2.5 mM ABTS as co-substrate. It was observed that the native r*Cvi*UPO and its T158A variant are inhibited by high H_2_O_2_ concentrations (Figure S4E), with inhibition constants (*k*_i_) of 5.6 mM and 1.8 mM, respectively (Table 1). The r*Cvi*UPO variants showed decreased catalytic efficiency for the enzyme-oxidizing substrate, due to 6- to 15-fold lower affinity for H_2_O_2_ than the native r*Cvi*UPO, with all the *K*_m_ values > 1 mM (native r*Cvi*UPO included). Therefore, the *k*_cat_ values for the different enzyme-reducing substrates would be often undervalued under the above conditions.

For this reason, second sets of kinetic curves and constants were obtained using 24 mM H_2_O_2_ (Figure S4F–I and Table S2, respectively) for better enzyme saturation. Under these conditions, increased catalytic efficiency of the simple and double variants could be observed. For F88A/T158A, up to 75-, 10- and 4-fold increases (with respect to native r*Cvi*UPO) were found for naphthalene, veratryl alcohol/ABTS, and benzyl alcohol, respectively. The broadest channel variant 6Ala showed improved affinity with all substrates, but often also lower reaction rates, resulting in lower catalytic efficiency.

Concerning oleic acid oxygenation by native r*Cvi*UPO and variants (Table 2), smaller differences in kinetic constants than those found for the other substrates were generally observed. Among them, it is worth mentioning the following: (i) a 50% increase of catalytic efficiency by the F88A/T158A variant, due to moderate improvements in both affinity and turnover; and (ii) over 5-fold reduction in catalytic efficiency by the F88A and 6Ala variants. 

The structural changes introduced in the new variants generated some catalytic improvements in oxygenation ability, particularly with the F88A/T158A variant compared with the native r*Cvi*UPO, as shown using naphthalene and oleic acid. These results indicate that this heme channel variant is a potentially interesting epoxidation biocatalyst, as described below. 

### 3.2. Fatty-Acid Oxygenation Patterns by rCviUPO and Heme-Channel Variants 

GC-MS analyses revealed different oxygenation products in the reactions of oleic, linoleic, and α-linolenic acids with r*Cvi*UPO and its F88A, T158A, F158A/T158A, and 6Ala variants (see chromatographic profiles in Figures S5–S7). The relative abundance of the different product classes—namely 9-epoxy, 12-epoxy, 15-epoxy, diepoxy, OH-/keto, and OH-epoxy derivatives—together with the conversion percentage and the epoxidation yield, are indicated in Table 3. In all cases, up to 88–99% conversions were attained with the native enzyme and its variants. Due to the native r*Cvi*UPO selectivity towards mono-epoxidation, its epoxidation yield decreased from oleic acid (71%) to α-linolenic acid (32%). However, the changes introduced in the mutated variants improved the epoxidation yields, reaching 93% with oleic acid (T158A), 90% with linoleic acid (F88A/T158A), and 60% with α-linolenic acid (F88A/T158A).

**Figure 3:**
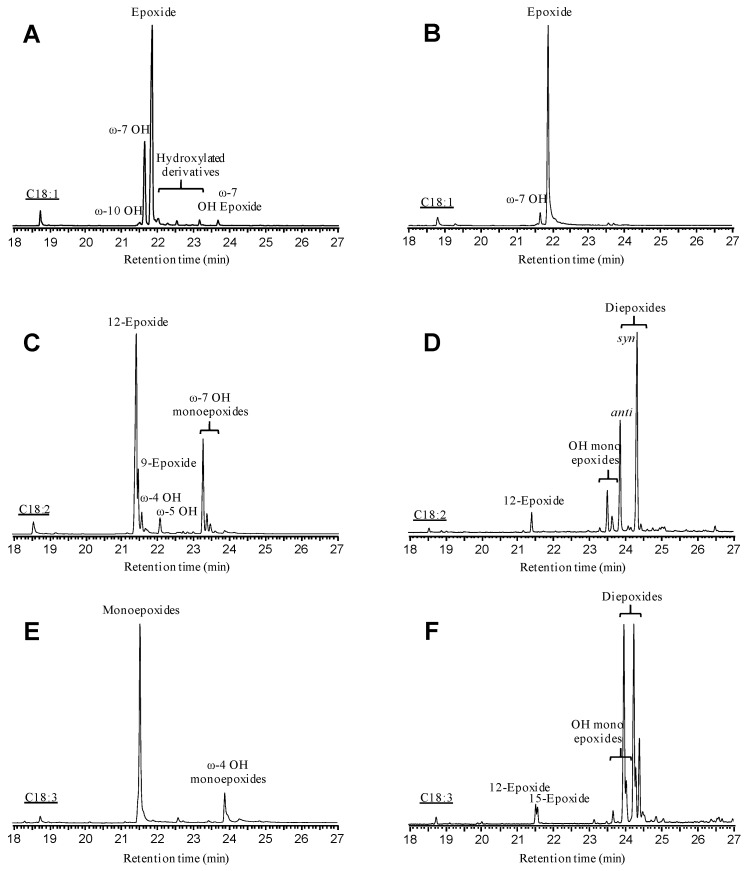
GC-MS profiles of selected epoxidation reactions of: (i) oleic acid (C18:1) treated with the 6Ala variant (**B**) compared with native r*Cvi*UPO (**A**), (ii) linoleic acid treated with the F88A/T158A variant (**D**) compared with the native r*Cvi*UPO (**C**), and (iii) α–linolenic acid treated with the F88A/T158A variant (**F**) compared with the native r*Cvi*UPO (**E**) (see Figures S5–S7 for the whole set of reactions). Mixtures containing 0.1 mM substrate, 0.25–0.40 µM enzyme, and 1.25 mM H_2_O_2_ were incubated for 30 min, extracted, and derivatized before GC-MS analysis.

Concerning the products, chromatograms in Figure 3 illustrate the most interesting reactions: (i) preferential epoxidation of oleic acid by the 6Ala variant (B) without the fatty acid hydroxylated derivatives (at the allylic position) formed by the native enzyme (A), and (ii) preferential diepoxidation (>80% of products) of linoleic and α–linolenic acids (D and F, respectively) by the F88A/T158A variant instead of the mono-epoxidation produced by the native enzyme (C and E, respectively). 

Finally, encouraged by the recently reported enantioselectivity of fatty acid mono-epoxidations with UPOs [24,25], reactions with oleic acid were carried out at a larger scale, and the epoxyoleic products were purified prior to chiral HPLC analysis. The 6Ala variant was selected due to its high regioselectivity to the epoxide, together with the F88A/T158A variant as the best UPO in terms of the overall epoxidation yield with all substrates (and native r*Cvi*UPO for comparative purposes). The results obtained (Figure S8) revealed only low enantioselectivity with the three enzymes (*ee* 0–40%), although an inversion of the configuration of the main enantiomer was produced between the F88A/T158A and 6Ala variants (70% *S*/*R* and 60% *R*/*S*, respectively).

### 3.3. Optimization of Hydrolyzed Sunflower Oil Epoxidation

After testing the r*Cvi*UPO variants on individual unsaturated fatty acids, the goal was to accomplish the epoxidation of hydrolyzed vegetable oil. For this purpose, a sunflower oil hydrolyzate was used as substrate, and several parameters—including substrate, enzyme, and H_2_O_2_ concentrations, among others—were optimized. 

**Figure 4:**
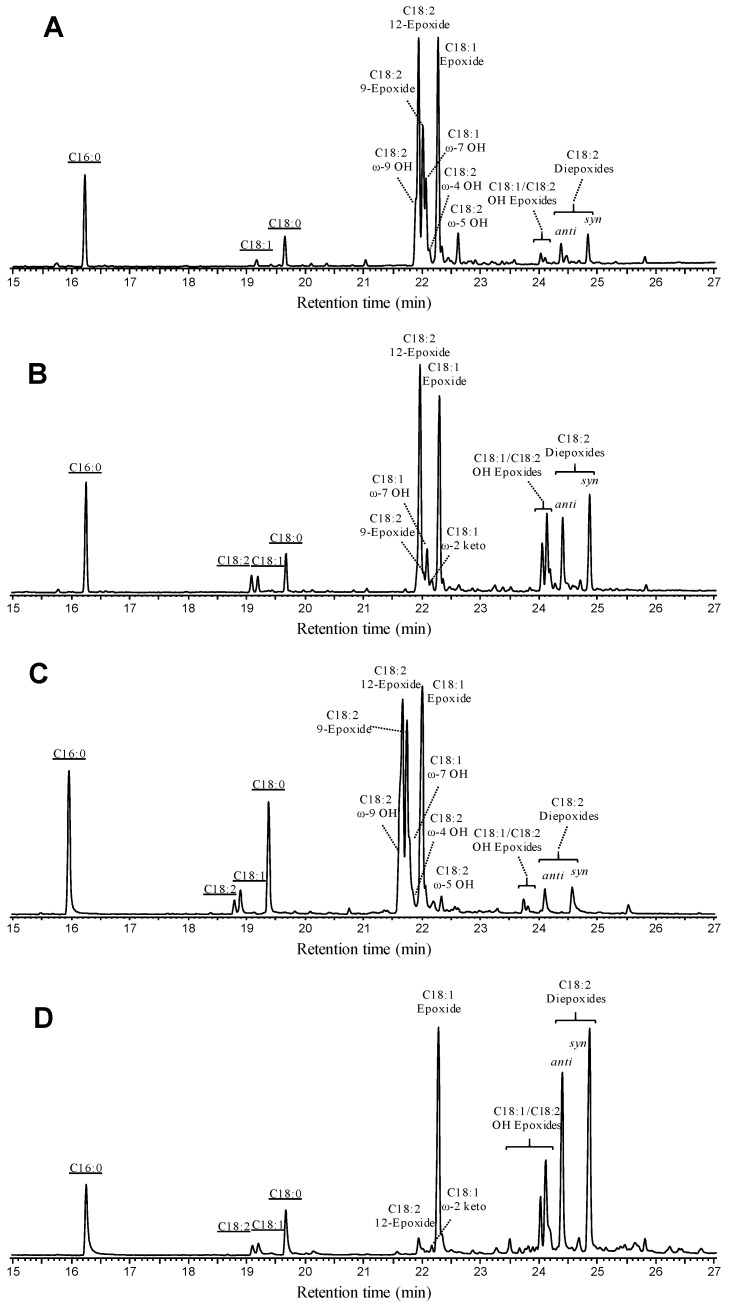
GC-MS profiles of UPO reactions with sunflower oil hydrolyzate. (**A**,**B**) Initial treatment of 0.1 mM oil hydrolyzate with 0.25 µM r*Cvi*UPO and its F88A/T158A variant, respectively, for 30 min in 50 mM phosphate (pH 7) containing 20% acetone and 1 mM H_2_O_2_. (**C**,**D**) Optimized treatment of 10 mM oil hydrolyzate with 25 µM r*Cvi*UPO and 50 µM F88A/T158A for 60 min in 50 mM phosphate buffer (pH 7) containing 30% acetone and 25 mM H_2_O_2_.

Firstly, reactions under the same conditions used with pure fatty acids were performed on hydrolyzed sunflower oil (0.1 mM) using native r*Cvi*UPO and its F88A/T158A variant (Figure 4A,B, respectively). Both enzymes were able to convert the oil unsaturated fatty acids with similar regioselectivity, forming (i) monoepoxides from oleic acid and linoleic acid (at 9 and 12 positions) as main products, (ii) two diepoxy isomers (*anti* and *syn*) from linoleic acid, more abundant in the F88A/T158A reaction; and (iii) small amounts of hydroxy- and hydroxy-epoxy derivatives of oleic acid and linoleic acids, together with some minor products.

Then, differences in the epoxidation yields were observed when varying the enzyme dose, using different substrate/enzyme (S/E) molar ratios. As shown in Table 4 (entries 1–6), increasing the r*Cvi*UPO dose did not result in improved epoxidation yield, but doubling the amount of F88A/T158A (0.5 μM, S/E ratio 200) yielded higher amounts of diepoxides from linoleic acid (the epoxidation of oleic acid was retained); consequently, a better epoxidation yield (72%) was attained. Nevertheless, a 1 μM dose of the double variant (S/E ratio 100) produced virtually the same epoxidation profile. Therefore, optimal 400 and 200 S/E ratios were fixed for further scale up with r*Cvi*UPO and its double variant, respectively.

However, when the substrate loading was increased to 5 mM (Table 4 entries 7–10), its conversion rate decayed with both enzymes within 30 min reactions. Therefore, an extension of the reaction time up to 60 min was needed to reverse the epoxidation performance with the F88A/T158A variant at the initial reaction level (entry 10). In fact, the reaction time becomes critical in UPO epoxidation of fatty acids when the substrate concentration is close to the solvent saturation [28].

Finally, with 10 mM substrate loading (Table 4 entries 11–17), the importance of the substrate solubilization was revealed by comparing the epoxidation yields with 20% and 30% acetone cosolvent. With both enzymes, 20% acetone resulted in low conversion and epoxidation yields (longer times did not improve the results). However, the complete solubilization of substrates using 30% acetone improved the epoxidation yield, especially with the double variant (entry 14) forming mono and diepoxide amounts similar to those observed in reactions with a lower substrate concentration.

With the enzyme dose, amount of co-solvent, and reaction time already optimized, assays were conducted to determine the lowest dose of H_2_O_2_ that enables the production of the highest epoxidation yield. As depicted in Table 4, the highest epoxidation yield (85%) was attained using only 1.7 equivalents of this oxidizer (entry 16), compared with the 6.8 (used in most previous assays) and 3.4 (entry 15) equivalents, while adding a stoichiometric amount of H_2_O_2_ (entry 17) led to a decrease in epoxidation yield (to only 44%).

The pattern of oxygenation products produced under optimized conditions (Figure 4C,D) was similar to that obtained previously, with linoleic acid diepoxides and oleic acid monoepoxide as the main products in the F88A/T158A reactions. No triepoxides were detected in any of the reactions, due to the low α–linolenic acid content of sunflower oil [37]. The above results reveal the remarkable potential of the r*Cvi*UPO double variant for epoxidizing sunflower oil hydrolyzate, demonstrating a much higher conversion yield than the native enzyme.

## 4. Conclusions

Opening the r*Cvi*UPO heme access channel allowed us to improve the epoxidation activity of this enzyme, selected for protein engineering in *E. coli*. In this way, the variant with the widest channel, after a sextuple mutation, yielded the highest epoxidation of oleic acid to the corresponding monoepoxide (with less than 5% of other oxygenation products). In contrast, a double variant (F88A/T158A) produced the best conversion of both linoleic and α–linolenic acids with diepoxides, of interest as crosslinking molecules, representing > 80% of products. Under the same conditions used for individual fatty acids, the double variant also converted hydrolyzed sunflower oil with a higher epoxidation yield than the native enzyme. Moreover, process optimization permitted us to increase (×100) the hydrolyzate concentration, epoxidizing 85% of double bonds after 1 h of reaction time with the mutated double variant. UPO engineering, strongly limited in the past by difficulties in heterologous expression of these enzymes in adequate hosts, is a requirement for developing the large repertoire of reactions of industrial relevance suggested in recent reviews [23,39,40,41,42,43,44,45] on an enzyme family of the highest interest for selective oxyfunctionalization reactions [46]. 

## Figures and Tables

**Table 1:** Kinetic constants for H_2_O_2_ reaction with native r*Cvi*UPO and variants.

	*K*_m_ (µM)	*k*_cat_/*K*_m_ (s^−1^·mM^−1^)	*k*_i_ (µM)
Native	1600 ± 468	374 ± 129	5580 ± 1800
F88A	16,300 ± 2900	29 ± 6	-
T158A	23,800 ± 13,700	127 ± 99	1790 ± 1000
F88A/T158A	9370 ± 880	55 ± 6	-
6Ala	14,800 ± 1600	0.7 ± 0.1	-

Measured in 100 mM acetate (pH 4) with 2.5 mM ABTS as reducing substrate. The high error values in the r*Cvi*UPO and T158A constants originate from the use of Equation (2) due to observed inhibition (Figure S4E) that, despite the good adjustments (R^2^ values of 0.988 and 0.997, respectively), has limited precision in predicting kinetic constants.

**Table 2:** Kinetic constants for oleic acid oxygenation by native r*Cvi*UPO and variants.

	*k*_cat_ (s^−1^)	*K*_m_ (µM)	*k*_cat_/*K*_m_ (s^−1^·mM^−1^)	*k*_i_ (µM)
Native	12.6 ± 0.3	58.8 ± 6.0	214 ± 53	-
F88A	10.5 ± 1.7	278.0 ± 49.0	38 ± 9	1120 ± 400
T158A	21.0 ± 1.1	89.0 ± 16.4	184 ± 35	-
F88A/T158A	16.5 ± 0.5	51.6 ± 5.4	319 ± 35	2900 ± 610
6Ala	16.6 ± 3.6	399.0 ± 94.6	42 ± 13	1360 ± 710

The kinetic constants were measured in 50 mM phosphate buffer (pH 7) in the presence of 24 mM H_2_O_2_.

**Table 3:** Fatty acid conversion, percentages of main products, and epoxidation yield (calculated with respect to the total number of double bonds present) in the reactions of 18-C fatty acids with native r*Cvi*UPO and four heme channel variants.

	Conversion			Products	(%)			Epoxidation
	(%)	15-Epoxy	12-Epoxy	9-Epoxy	Diepoxy	OH/Keto	OH-Epoxy	Yield (%)
C18:1								
Native	96	-	-	71	-	28	1	71
F88A	97	-	-	69	-	6	25	91
T158A	98	-	-	87	-	5	8	93
F88A/T158A	95	-	-	63	-	13	24	82
6Ala	96	-	-	96	-	4	-	92
C18:2								
Native	97	-	56	10	-	8	26	45
F88A	98	-	15	2	46	12	25	66
T158A	88	-	23	17	29	27	4	43
F88A/T158A	99	-	4	-	81	-	15	90
6Ala	99	-	-	25	64	-	11	81
C18:3								
Native	96	77	6	2	-	-	15	32
F88A	98	16	6	4	53	8	13	47
T158A	98	26	30	17	3	20	3	27
F88A/T158A	99	2	3	-	82	-	13	60
6Ala	99	17	35	16	10	16	6	47

The products from 30 min reactions of 0.25 µM or 0.40 µM enzyme with C18:1/C18:3 and C18:2, respectively, in 50 mM phosphate buffer (pH 7) containing 20% acetone, were extracted with methyl *tert*-butyl ether and analyzed by GC-MS as trimethylsilyl derivatives.

**Table 4:** Optimizing epoxidation yield (as percentage of the number of total double bonds present) in treatment of sunflower oil hydrolyzate with the selected r*Cvi*UPO variant (F88A/T158A) compared with native enzyme, by varying reaction parameters—such as substrate loading (mM), enzyme dose (µM), substrate/enzyme (S/E) molar ratio, acetone content (*v*/*v* %), H_2_O_2_ dose (mM and equivalents per fatty-acid double bond), and time (min)—under different combinations (entries 1–17).

Entry	Preparation	Substrate, mM	Enzyme, µM (S/E Ratio)	Acetone, %	H_2_O_2_, mM (Equiv)	Time, min	Epoxidation Yield, %
1	native	0.1	0.25 (400)	20	1 (6.8)	30	54
2	native	0.1	0.5 (200)	20	1 (6.8)	30	52
3	native	0.1	1 (100)	20	1 (6.8)	30	53
4	F88A/T158A	0.1	0.25 (400)	20	1 (6.8)	30	59
5	F88A/T158A	0.1	0.5 (200)	20	1 (6.8)	30	72
6	F88A/T158A	0.1	1 (100)	20	1 (6.8)	30	75
7	native	5	12.5 (400)	20	50 (6.8)	30	8
8	native	5	12.5 (400)	20	50 (6.8)	60	28
9	F88A/T158A	5	25 (200)	20	50 (6.8)	30	35
10	F88A/T158A	5	25 (200)	20	50 (6.8)	60	84
11	native	10	25 (400)	20	100 (6.8)	60	37
12	native	10	25 (400)	30	100 (6.8)	60	56
13	F88A/T158A	10	50 (200)	20	100 (6.8)	60	40
14	F88A/T158A	10	50 (200)	30	100 (6.8)	60	79
15	F88A/T158A	10	50 (200)	30	50 (3.4)	60	78
16	F88A/T158A	10	50 (200)	30	25 (1.7)	60	85
17	F88A/T158A	10	50 (200)	30	15 (1.0)	60	44

## Data Availability

All data underlying this article are available in the main publication and in its Supplementary Material online.

## Supplementary Materials

The following supporting information can be downloaded at https://www.mdpi.com/article/10.3390/antiox11050915/s1. Kinetic constants for substrate oxidation by native r*Cvi*UPO and variants in the presence of 1 mM and 24 mM H_2_O_2_ (Table S1 and Table S2, respectively); SDS-PAGE of purified native rCviUPO and variants (Figure S1); UV–visible spectra of resting states and CO complexes of rCviUPO and variants (Figure S2); Effect of pH on the oxidation of different substrates (Figure S3); Kinetic curves for different UPO reducing substrates and H_2_O_2_ (Figure S4); GC-MS analyses of oleic, linoleic, and α–linolenic acids reactions with CviUPO and four heme channel variants (Figure S5, Figure S6 and S7, respectively); and Chiral HPLC analysis of oleic acid epoxide (Figure S8).
