# Supplementary material for: Engineering Collariella virescens Peroxygenase for Epoxides Production from Vegetable Oil
Source: Antioxidants (Basel). 2022 May 6;11(5):915. doi: 10.3390/antiox11050915 (PMC9137900; doi:10.3390/antiox11050915)
Supplement: Supplementary file 1 [file antioxidants-11-00915-s001.zip › antioxidants-1683917-supplementary.pdf]

antioxidants

MDPI

Article

SUPPLEMENTARY MATERIALS

Engineering *Collariella virescens* Peroxygenase for Epoxides Production from Vegetable Oil

Dolores Linde [1,†], Alejandro González-Benjumea [2,†], Carmen Aranda [3], Juan Carro [1], Ana Gutiérrez [2] and Angel T. Martínez [1,*]

[1] Centro de Investigaciones Biológicas "Margarita Salas" (CIB), CSIC, E-28040 Madrid, Spain; lolalinde@cib.csic.es (D.L.); jcarro@cib.csic.es (J.C.)
[2] Instituto de Recursos Naturales y Agrobiología de Sevilla (IRNAS), CSIC, E-41012 Seville, Spain; a.g.benjumea@irnas.csic.es (A.G.-B.); anagu@irnase.csic.es (A.G.)
[3] Johnson Matthey, Cambridge Science Park U260, Cambridge CB4 0FP, UK; carmen.aranda@matthey.com
* Correspondence: atmartinez@cib.csic.es; Tel.: +34-918373112
† These authors contributed equally to this work.

This Supplementary Materials includes: Kinetic constants for substrate oxidation by native r*Cvi*UPO and variants in the presence of 1 mM and 24 mM H_2O_2 (**Tables S1** and **S2,** respectively); SDS-PAGE of purified native r*Cvi*UPO and variants (**Figure S1**); UV-visible spectra of resting states and CO complexes of r*Cvi*UPO and variants (**Figure S2**); Effect of pH on the oxidation of different substrates (**Figure S3**); Kinetic curves for different UPO reducing substrates and H_2O_2 (**Figure S4**); GC-MS analyses of oleic acid, linoleic, and α–linolenic acids reactions with *Cvi*UPO and four heme channel variants (**Figures S5-S7**, respectively); and Chiral HPLC analysis of oleic acid epoxide (**Figure S8**).

Table S1. Kinetic constants for substrate (veratryl and benzyl alcohols, naphthalene and ABTS) oxidation by the native *Cvi*UPO and variants (F88A, T158A, F88A/T158A and 6Ala) in the presence of 1 mM H_2O_2.

	k_{cat} (s^{-1})	K_m (μM)	k_{cat}/K_m (s^{-1} mM^{-1})	ki(μM)	nH
Veratryl alcohol					
Native	2.24 ± 0.03	2,940± 159	0.75 ± 0.03	-	-
F88A	2.65 ± 0.19	1,050 ± 159	2.51 ± 0.12	-	-
T158A	1.51 ± 0.03	820 ± 100	1.84 ± 0.19	-	-
F88A/T158A	2.40 ± 0.10	1,720 ± 340	1.38 ± 0.23	-	-
6Ala	0	-	0	-	-
Naphthalene					
Native	1.09 ± 0.07	450 ± 70	2.42 ± 0.24	-	-
F88A	3.68 ± 0.17	435 ± 563	8.47 ± 0.74	-	-
T158A	1.28 ± 0.04	180 ± 14	7.17 ± 0.42	-	-
F88A/T158A	1.62 ± 0.05	100 ± 12	16.20 ± 1.50	-	-
6Ala	0	-	0	-	-
ABTS					
Native	157.0 ± 3.0	239 ± 8	656 ± 26	7,860 ± 680	-
F88A	16.3 ± 0.8	48 ± 7	334 ± 54	98,400 ± 11,200	-
T158A	163.0 ± 24.0	260 ± 60	627 ± 171	811 ± 204	-
F88A/T158A	5.3 ± 0.3	7 ± 2	768 ± 197	-	-
6Ala	5.2 ± 0.1	83 ± 11	62 ± 7	-	-
Benzyl alcohol					
Native	63.0 ± 2.3	7,050 ± 570	8.92 ± 0.42	-	1.6 ± 0.1
F88A	20.1 ± 1.0	5,420 ± 91	3.71 ± 0.66	47,600 ± 9,100	-
T158A	128.1 ± 6.6	8,970 ± 1460	14.28 ± 2.37	-	1.5 ± 0.2
F88A/T158A	8.5 ± 0.5	809 ± 138	10.70 ± 1.89	32,400 ± 6,100	-
6Ala	9.6 ± 1.5	4,300 ± 2400	2.20 ± 1.60	-	-

The kinetic constants were measured in 100 mM acetate (veratryl alcohol and ABTS), 100 mM Tris (benzyl alcohol) or 100 mM tartrate (naphthalene) at the optimal pH for each enzyme-substrate couple

Table S2. Kinetic constants for substrate (veratryl and benzyl alcohols, naphthalene, and ABTS) oxidation by native r*Cvi*UPO and variants (F88A, T158A, F88A/T158A and 6Ala) in the presence of 24 mM H_2O_2.

	k_{cat} (s^{-1})	K_m (μM)	k_{cat}/K_m ($s^{-1}\cdot mM^{-1}$)	k_i (μM)
Veratryl alcohol				
Native	5.7 ± 0.2	20,800 ± 2,300	0.27 ± 0.03	-
F88A	11.1 ± 0.3	4,740 ± 480	2.34 ± 0.24	-
T158A	6.5 ± 0.4	37,500 ± 5,500	0.17 ± 0.02	-
F88A/T158A	43.5 ± 1.8	15,000 ± 2,000	2.89 ± 0.39	-
6Ala	3.7 ± 0.3	3,590 ± 1,040	1.03 ± 0.31	-
Naphthalene				
Native	2.3 ± 0.3	648 ± 235	3.5 ± 0.8	-
F88A	13.9 ± 0.91	239 ± 51	58.1 ± 12.9	-
T158A	1.8 ± 0.1	160 ± 37.6	11.2 ± 2.7	-
F88A/T158A	39.8 ± 2.4	151 ± 33	263.0 ± 38.0	-
6Ala	6.1 ± 0.7	360 ± 123	16.9 ± 6.1	-
ABTS				
Native	237 ± 19	576 ± 150	411 ± 86.8	-
F88A	630 ± 54	225 ± 44	2,800 ± 597	6,610 ± 1,900
T158A	200 ± 8	590 ± 76	339 ± 45.7	-
F88A/T158A	660 ± 23	153 ± 23	4,310 ± 670	-
6Ala	12 ± 1	391 ± 42	30 ± 3	-
Benzyl alcohol				
Native	59 ± 2	8,020 ± 1,030	7.3 ± 1.0	-
F88A	210 ± 41	25,700 ± 10,500	8.2 ± 3.7	-
T158A	380 ± 51	45,300 ± 10,500	8.4 ± 2.2	-
F88A/T158A	297 ± 31	9,490 ± 2,840	31.3 ± 9.9	-
6Ala	16 ± 1	3,850 ± 220	4.1 ± 0.3	-

The kinetics constants were measured in 100 mM acetate (veratryl alcohol and ABTS), 100 mM Tris (benzyl alcohol) or 100 mM tartrate (naphthalene) at the optimal pH for each enzyme-substrate couple.

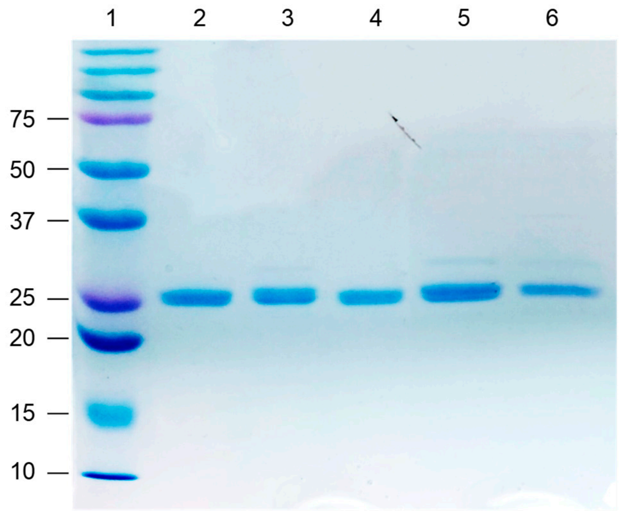

Figure S1. SDS-PAGE of purified native r*Cvi*UPO (*lane 2*) and variants F88A (*lane 3*), T158A (*lane 4*), F88A/T158A (*lane 5*) and 6Ala (*lane 6*). Molecular-mass markers are indicated in kDa (*lane 1*).

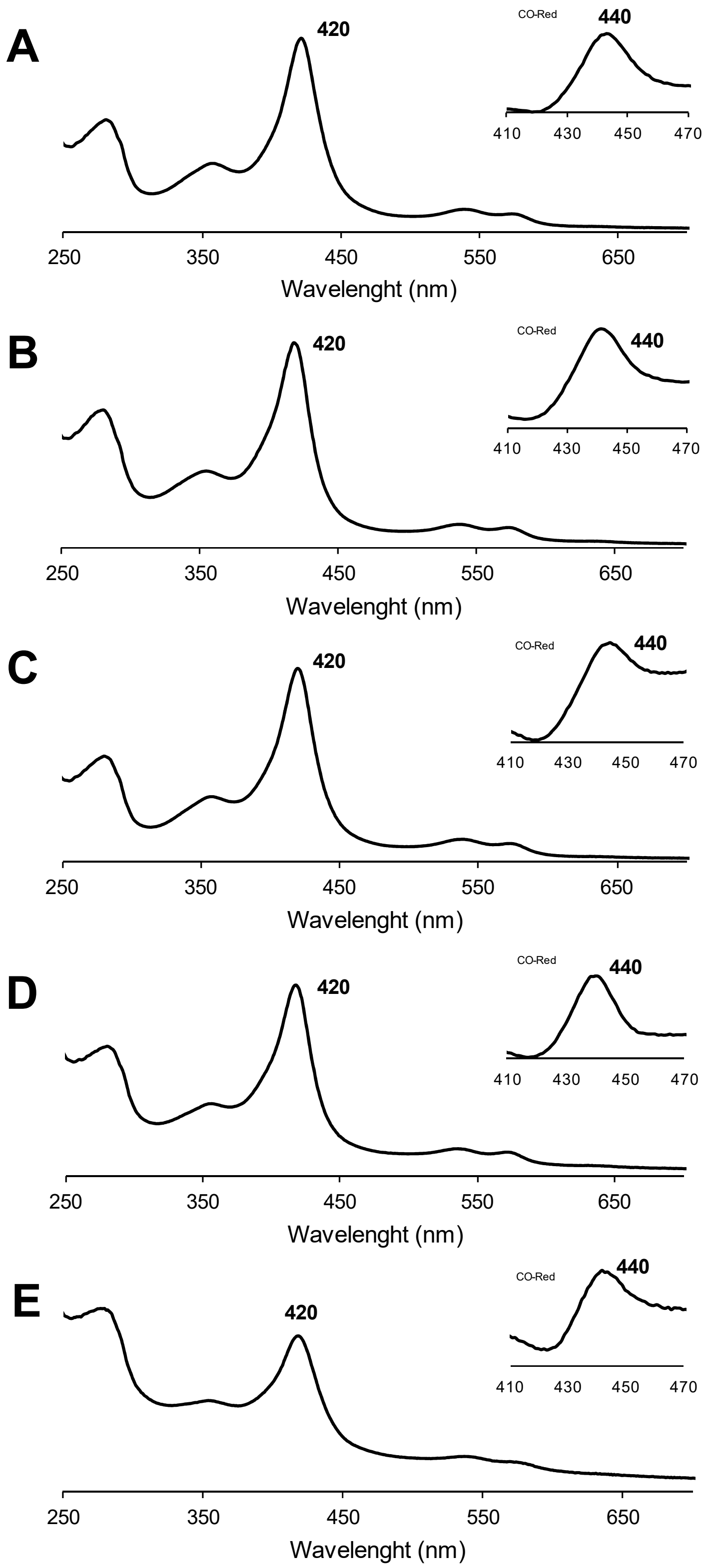

Figure S2. UV-visible spectra of resting states (main panels) and CO complexes (insets) of native r*Cvi*UPO (**A**) and its F88A (**B**), T158A (**C**), F88A/T158A (**D**) and 6Ala (**E**) heme-channel variants. The resting-state Soret band (around 420 nm in the main panels) is characteristically displaced (at around 440 nm), in the complexes between the reduced enzyme and CO, as shown in the difference spectra included in the insets (obtained by subtracting the spectrum of the reduced enzyme).

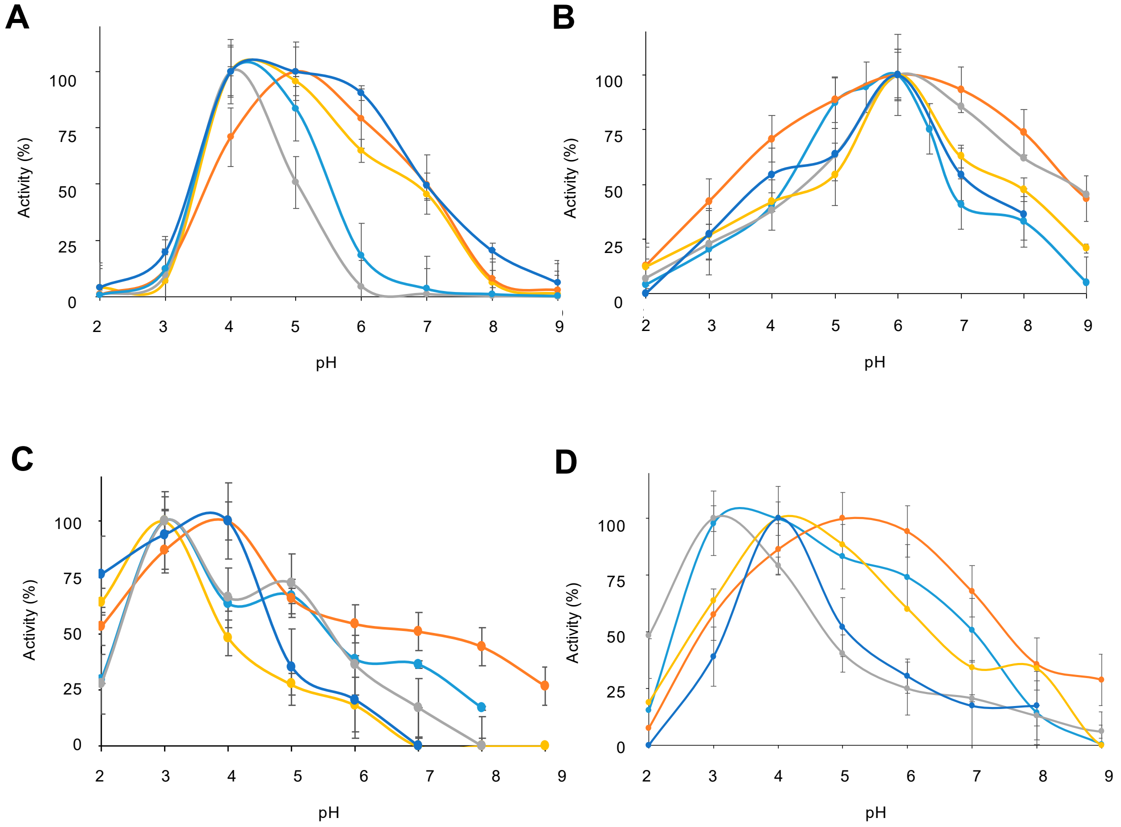

Figure S3. Effect of pH on oxidation of four UPO substrates. Relative activity of native r*Cvi*UPO (*cyan*) and its F88A (*orange*), T158A (*gray*), F88A/T158A (*yelow*) and 6Ala (*dark blue*) variants oxidizing 2 mM ABTS (**A**), 10 mM benzyl alcohol (**B**), 1 mM naphthalene (**C**) and 10 mM veratryl alcohol (**D**) at different pH values (measured in 50 mM Britton-Robinson buffer, pH 2-9). Bars represent the standard deviations of the means of three measurements.

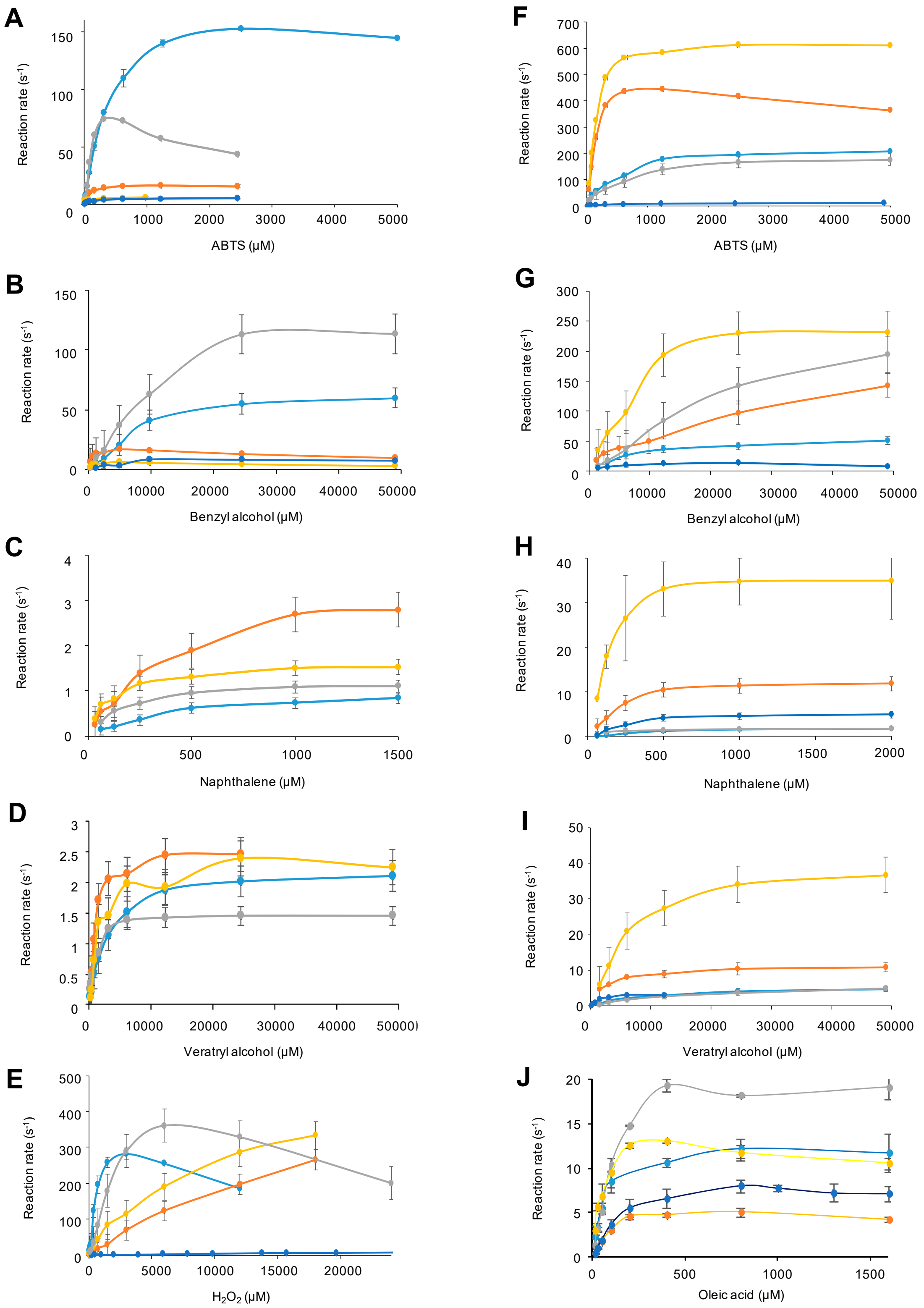

Figure S4. Kinetic curves for UPO reducing substrates and H_2O_2. The reaction rates of native r*Cvi*UPO (*cyan*) and its F88A (*orange*), T158A (*gray*), F88A/T158A (*yelow*) and 6Ala (*dark blue*) variants with different concentrations of ABTS (**A**,**F**), benzyl alcohol (**B**,**G**), naphthalene (**C**,**H**), veratryl alcohol (**D**,**I**), H_2O_2 (**E**) and oleic acid (**J**) were measured using 1 mM (**A-D**) or 24 mM (**F-J**) H_2O_2, and 2.5 mM ABTS (**E**) as cosubstrate. Bars represent the standard deviations of the means of three measurements.

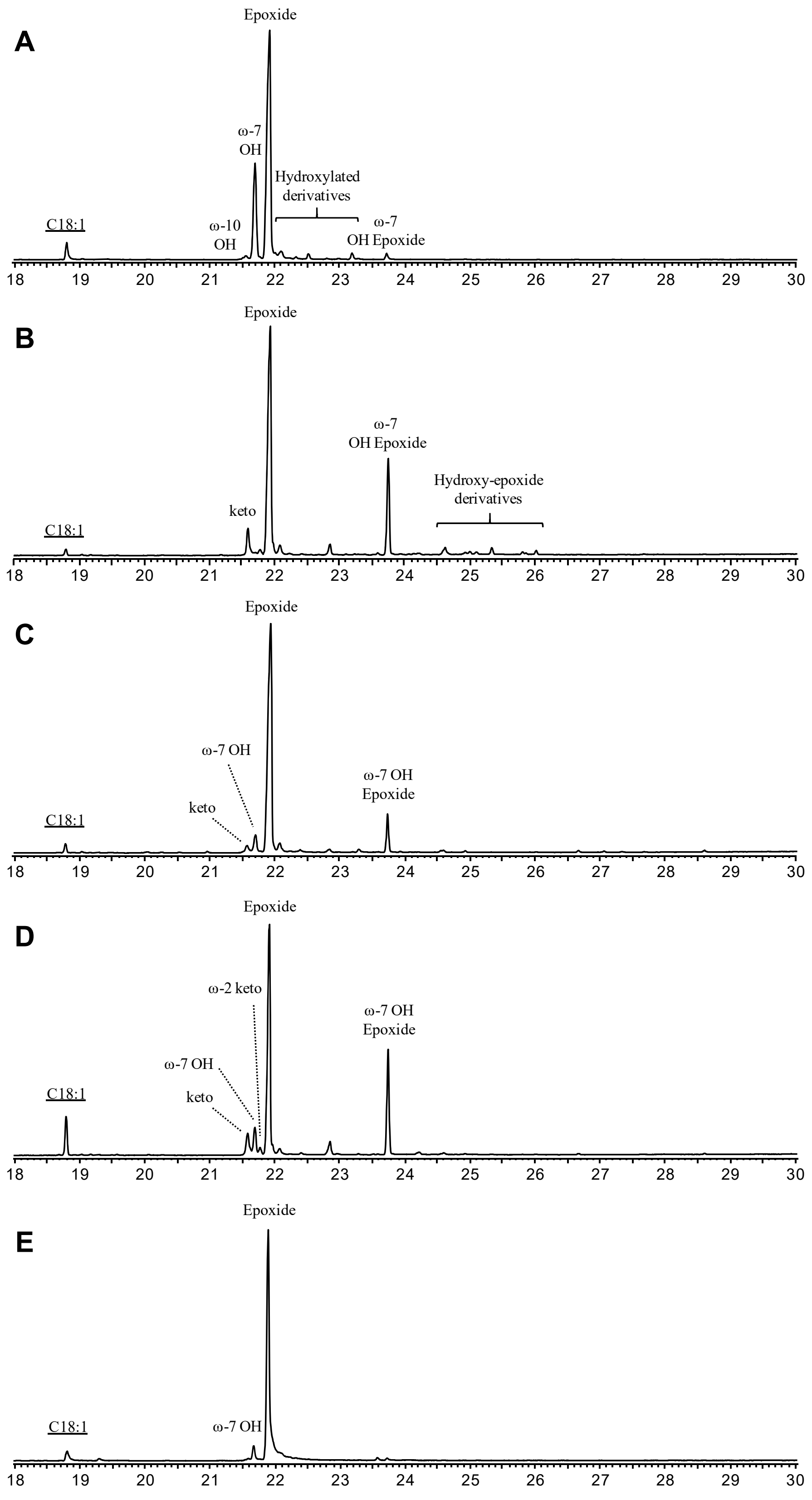

Figure S5. GC-MS analysis of oleic acid (C18:1) reactions with *Cvi*UPO (**A**) and its F88A (**B**), T158A (**C**), F88A/T158A (**D**) and 6Ala (**E**) variants. Reaction mixtures containing 0.1 mM substrate, 1.4 μM enzyme and 1.25 mM H_2O_2 were incubated for 30 min, extracted, and derivatized before GC-MS analysis.

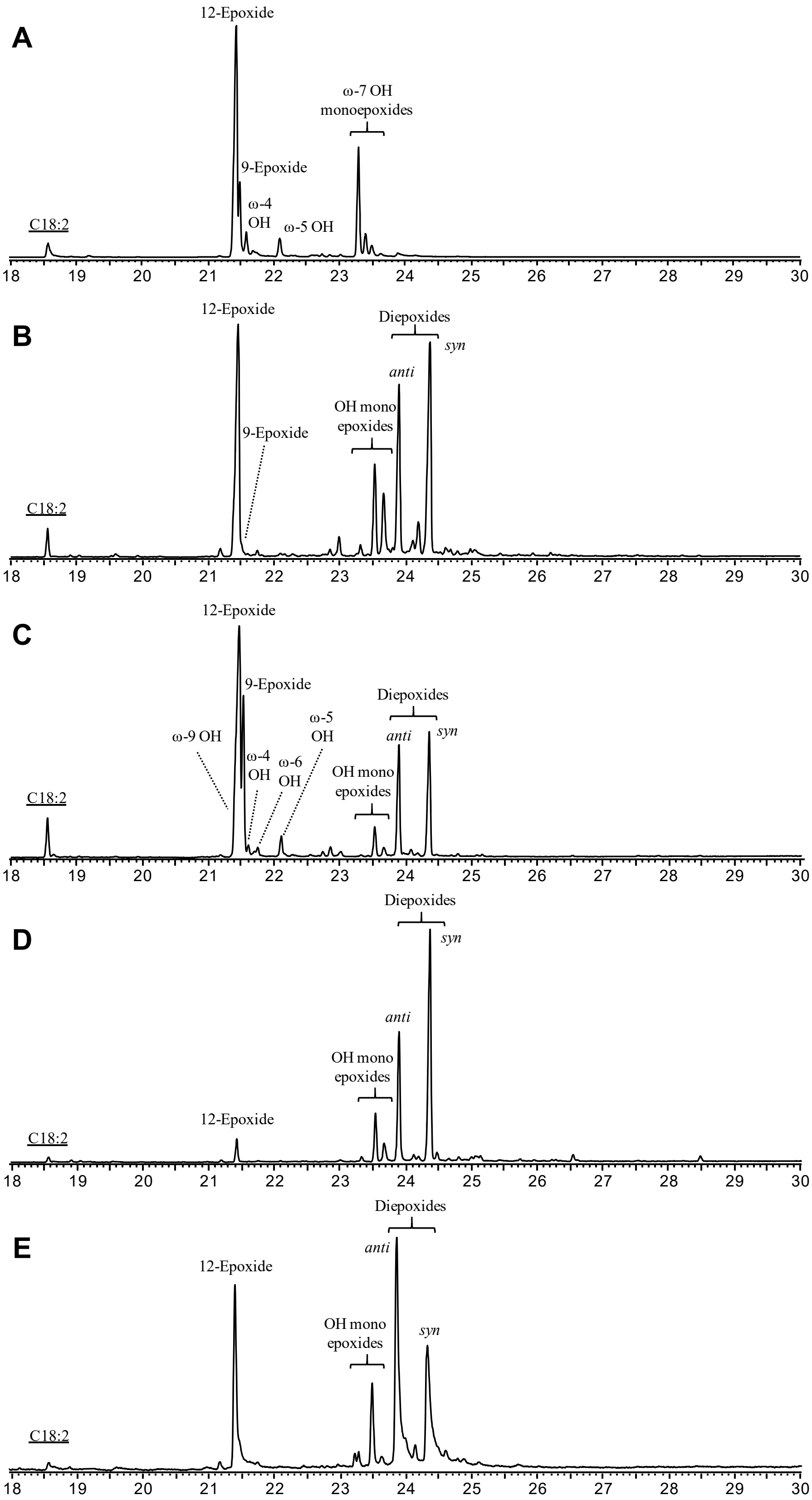

Figure S6. GC-MS analysis of linoleic acid (C18:2) reactions with *Cvi*UPO (**A**) and its F88A (**B**), T158A (**C**), F88A/T158A (**D**) and 6Ala (**E**) variants. Reaction mixtures containing 0.1 mM substrate, 1.4 µM enzyme and 1.25 mM H_2O_2 were incubated for 30 min, extracted, and derivatized before GC-MS analysis.

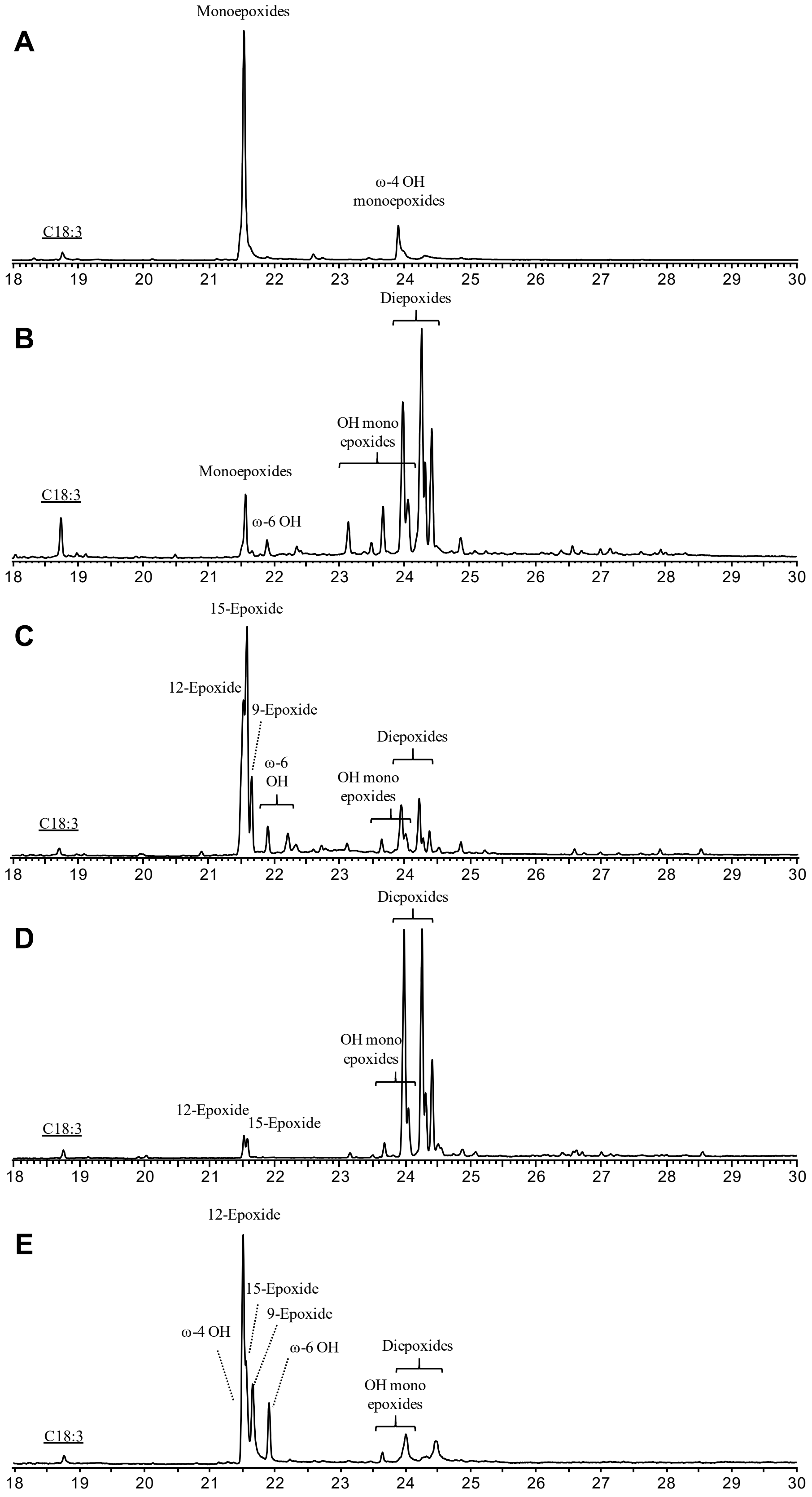

Figure S7. GC-MS analysis of α-linolenic acid (C18:3) reactions with *Cvi*UPO (**A**) and its F88A (**B**), T158A (**C**), F88A/T158A (**D**) and 6Ala (**E**) variants. Reaction mixtures containing 0.1 mM substrate, 1.4 µM enzyme and 1.25 mM H_2O_2 were incubated for 30 min, extracted, and derivatized before GC-MS analysis.

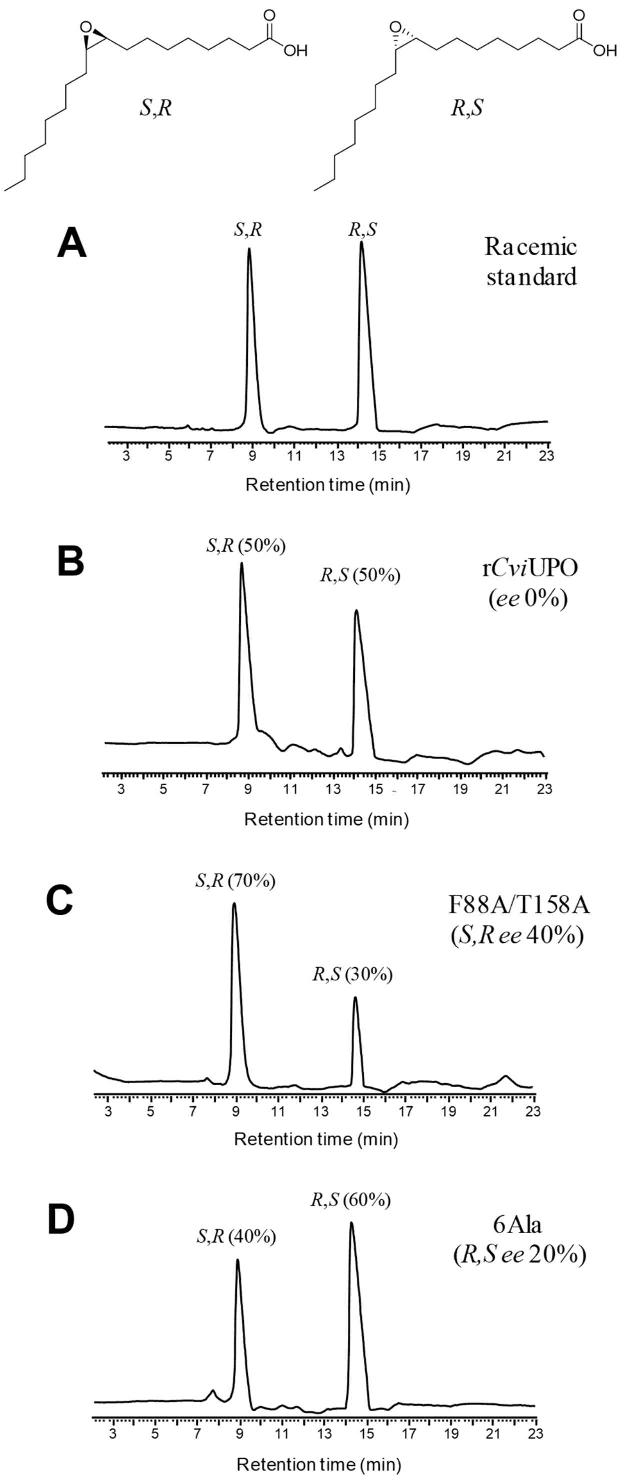

Figure S8. Chiral HPLC analysis of oleic acid epoxidation with native r*Cvi*UPO (**B**) and its F88A/T158A (**C**) and 6Ala (**D**) variants, compared with a racemic epoxyoleic acid standard (**A**), with indication of the relative abundance of the *S,R* and *R,S* enantiomers and the resulting *ee* values.
